# Insertion of Carbenes into Deprotonated *nido*-Undecaborane, B_11_H_13_(2-)

**DOI:** 10.3390/molecules24203779

**Published:** 2019-10-21

**Authors:** Jacek Pecyna, Igor Rončević, Josef Michl

**Affiliations:** 1 Department of Chemistry, University of Colorado, Boulder, CO 80309-0215, USA; Jacek.Pecyna@colorado.edu; 2 Institute of Organic Chemistry and Biochemistry Academy of Sciences of the Czech Republic, Flemingovo nám. 2, 16610 Prague 6, Czech Republic; Igor.roncevic@uochb.cas.cz

**Keywords:** carboranes, [*closo*-1-CB_11_H_12_]^−^ anion, difluorocarbene

## Abstract

We have examined the insertion of carbenes carrying leaving groups into the [*nido*-B_11_H_13_]^2−^ dianion to form the [*closo*-1-CB_11_H_12_]^−^ anion. The best procedure uses CF_3_SiMe_3_ and LiCl as the source of CF_2_. It is simple, convenient and scalable and proceeds with 70–90% yield. Density functional calculations have been used to develop a mechanistic proposal that accounts for the different behavior of CF_2_, requiring only one equivalent of base for successful conversion of Na[*nido*-B_11_H_14_]^−^ to [*closo*-1-CB_11_H_12_]^−^, and CCl_2_ and CBr_2_, which require more.

## 1. Introduction

The [*closo*-1-CB_11_H_12_]^−^ anion [1] (**1**) is a member of a broad class of compounds called carboranes [2]. They are derived from three-dimensional polyhedral boron hydrides in which at least at one vertex, a boron atom has been replaced with a carbon atom. These unusual structures are characterized by high thermal and chemical stability attributable to σ-electron delocalization. The monocarba-*closo*-dodecaborate(−) anion and its derivatives are of special interest as they are one of the most weakly-coordinating anions [3,4] and have a high positive redox potential [5]. The solubility of their salts in water and the possibility of functionalization at boron and carbon vertices make them suitable agents for boron neutron capture therapy [6]. The anion **1** has also been found to be an effective building block for highly polar liquid crystals [7,8,9] and functionalized carbaborates serve as counterions in ionic liquids [10,11].

**Scheme 1:**
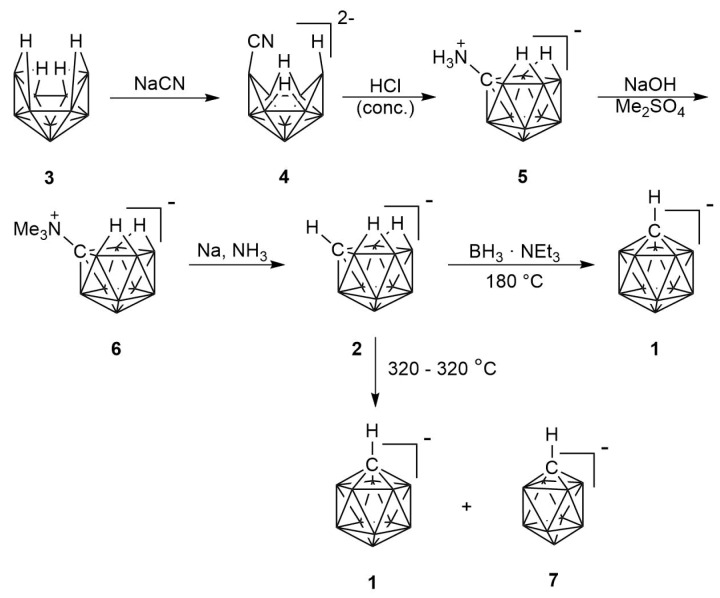
Synthesis of **1** by Knoth.

Several methods of synthesis of cluster **1** are known and each has its advantages. The first preparation (Scheme 1) was reported by Knoth [12,13] and relied on thermolysis of a salt of the [*nido*-7-CB_10_H_13_]^−^ anion (**2**). The precursor **2** was generated from decaborane, B_10_H_14_ (**3**) by treatment with sodium cyanide to give [*arachno*-6-CN-B_10_H_13_]^2−^ (**4**), which was then hydrolyzed using HCl to yield [*nido*-7-H_3_N-7-CB_10_H_12_] (**5**). The zwitterion **5** was methylated with Me_2_SO_4_ to give [*nido*-7-Me_3_N-7-CB_10_H_13_]^−^ (**6**), which was then deaminated with sodium in liquid ammonia to give **2**. Thermolysis yielded a mixture of **1** and [*closo*-1-CB_9_H_10_]^−^ (**7**). Treatment of **2** with BH_3_·NEt_3_ also led to the formation of **1**. 

**Scheme 2:**
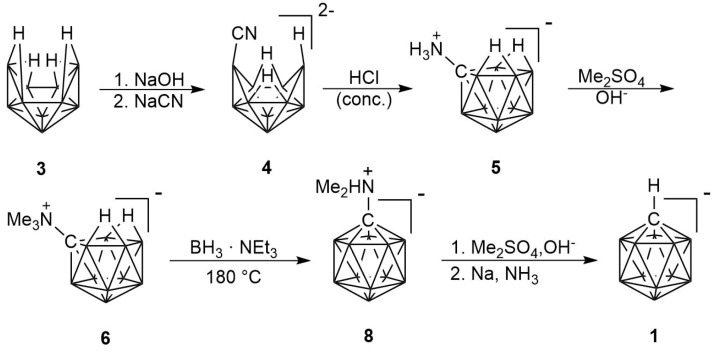
Heřmánek’s synthesis of **1** [14].

The method was modified and made somewhat safer by Heřmánek [14] (Scheme 2).

The synthesis still starts with decaborane. Only one equivalent of sodium cyanide is required to convert **3** into **4** and HCl is used in place of an ion exchange resin. Further steps include methylation of **5**, boron vertex insertion into **6**, remethylation of the amino group in **8** and deamination using sodium.

**Scheme 3:**
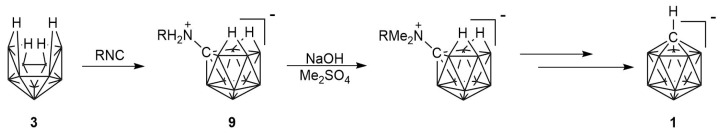
Preparation of **1** by Fox and Hughes [15].

The method developed by Fox and Hughes [15] eliminated the use of the toxic cyanide and took advantage of the facile conversion of **3** to **9** with isocyanides, similar to that with cyanide. Further steps resemble those in Knoth’s and Heřmánek’s methods (Scheme 3). 

**Scheme 4:**
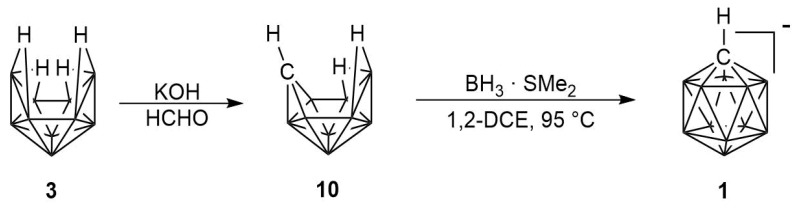
Kennedy’s preparation of **1** [16].

A more convenient route, which still requires the use of decaborane (**3**), was developed by Kennedy [16]. In this process, **3** is converted into [*arachno*-6-CB_9_H_14_]^−^ (**10**), which then undergoes a double boron vertex insertion to yield the desired **1** in 65% overall yield (Scheme 4).

Nowadays, this route to **1** is the most commonly used because it is simple and involves only two steps. However, a very large excess of BH_3_ · Me_2_S and three to four days of heating are required.

**Scheme 5:**
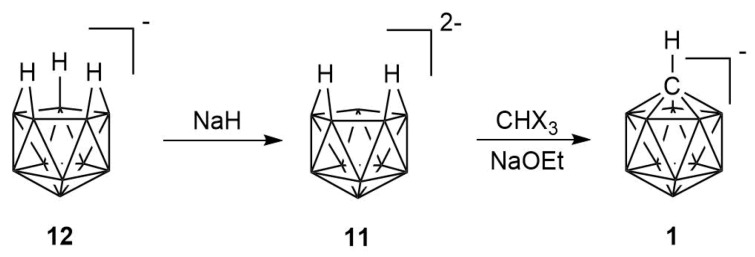
Michl’s synthesis of **1** [17].

A very different approach to the synthesis of **1** was taken by Michl and collaborators [17]. Instead of the expensive decaborane, the method started with the [*nido*-B_11_H_13_]^2−^ dianion (**11**) obtained by deprotonation of [*nido*-B_11_H_14_]^−^ (**12**), which was secured in one step from the inexpensive NaBH_4_ and BF_3_·Et_2_O [18] and inserted a dihalocarbene in a single reaction step (Scheme 5).

Both chloroform and bromoform were used as a carbene source. The drawbacks of the method are a very low yield, typically 20–25% (the highest achieved yield was 40%), poor scalability and the formation of various byproducts, the major ones being 2-ethoxylated and 2-halogenated **1** and [*nido*-7-OH-B_11_H_13_]^−^, which is formed from the product of oxidation, [*closo*-B_11_H_11_]^2−^, upon acidic workup. An attempt to use iodoform led only to the oxidation product.

Over the years, we have attempted to improve the yield of **1** by varying the nature of the carbene and the reaction conditions extensively. Presently, we report the details of our investigations, which resulted in a convenient procedure that affords a nearly quantitative yield. Our results are accompanied by a computational study of the mechanism. The overall yield of the easy two-step preparation of **1** from NaBH_4_ and BF_3_·Et_2_O now is 45–60%, making this desirable anion inexpensively accessible.

Surprisingly to us, the key to success was to use difluorocarbene. The same discovery was made independently by Leito and collaborators [19], who used a pressure vessel and reported their results earlier this year. The use of an initiator [20,21] for the formation of difluorocarbene from (trifluoromethyl)trimethylsilane in our procedure allowed us to work under conditions that are more convenient and we avoid the use of a glove-box and of pressure.

## 2. Results

### 2.1. Synthesis

Our initial extensive attempts to optimize and scale up the previously reported conditions for insertion of dihalocarbenes into **11**, using haloforms as the source of dichlorocarbene and dibromocarbene, proved unsuccessful. We turned to diethyl (dichloromethyl)phosphonate and tetraethyl (chloromethylene)bisphosphonate as the carbene source (Table 1, entries 1 and 2). The former was found to be effective in yielding **1** in 65% yield. The latter gave a 50% yield of the desired product, contaminated with significant amounts of the oxidation product, [*nido*-7-OH-B_11_H_13_]^−^. Reactions using the phosphonates were reproducible but poorly scalable. Rh-catalyzed reaction to generate a carbene from tetraethyl diphosphonodiazomethane only resulted in a complex mixture of unidentified products (Table 1, entry 3).

Attempts to obtain **1** with dichlorocarbene to be generated from sodium trichloroacetate (Table 1, entry 4) or with difluorocarbene, whose generation from sodium chlorodifluoroacetate was sought similarly (Table 1, entries 5 and 6), were unsuccessful. The high temperature required for carbene generation caused cluster decomposition and poor recovery of the starting material. Carefully dried lithium trifluoroacetate also proved not to be a good source of difluorocarbene at temperatures tolerated by the boron cluster.

Attempts using trimethyl(trifluoromethyl)silane as difluorocarbene source and NBu_4_Br as the initiator (Table 2, entries 1, 3–5) gave the desired product in high yields but the degree of conversion was poorly reproducible. Further attempts to modify the experimental conditions to yield **1** gave only the starting material or a mixture of the starting material and the desired product (Table 2, entries 2, 6–8). Interestingly, lowering the reaction temperature to 70 °C resulted mostly in the formation of the oxidation product, [*nido*-7-OH-B_11_H_13_]^−^ (Table 2, entry 8).

The addition of various lithium halides as a co-initiator for the reaction of **11** with TMS-CF_3_ proved to be successful in making the reaction reproducible. The desired product was obtained in 94% and 78% yield (Table 3, entry 1) using a combination of LiCl and TBAB. Two more runs using the same conditions gave **1** in 78% yield. Interestingly, changing the reaction solvent to 1,2-dimethoxyethane (higher boiling point) appeared to have a significant effect on the reaction outcome. Starting material recovery was poor and cluster decomposition was observed (Table 3 entries 2, 3 and 5). Increasing the volume of THF (Table 3, entry 4), changing the ratio of LiCl to TBAB (Table 3, entry 6) or increasing the number of equivalents of TMS-CF_3_ (Table 3, entry 7) did not improve the yield of **1**. The product was obtained in 69% yield when higher concentration of the dianion was used (Table 3, entry 8). Lowering the reaction temperature to 60 °C (Table 3, entry 9) also decreased the yield of **1**. Replacement of LiCl with LiBr (Table 3, entries 10 and 12) or LiI (Table 3, entry 11) in a combination with TBAB produced **1** in lower yields. The highest reproducible yield, 78%, based on three runs was achieved when TMS-CF_3_ (2.5 equiv) was used in the presence of LiCl (15 mol%) as the initiator in refluxing THF (10 mL) (Table 3, entry 14). A combination of LiCl and LiBr (Table 3, entry 13) afforded **1** in a comparable yield. Doubling the number of equivalents of TMS-CF_3_ (Table 3, entry 15) did not improve the yield. A simultaneous increase of the amount of TMS-CF_3_ and the initiator (Table 3, entries 16 and 17) surprisingly gave **1** in lower yields. When lithium bromide was employed as the initiator in place of lithium chloride (Table 3, entry 18), the desired product was afforded in a slightly lower yield (72%). Lowering the reaction temperature by a few degrees and using lithium bromide (Table 3, entry 19) caused a significant decrease in the yield (45%). That result is consistent with lower yields, 54% of **1**, when a lower temperature of 50 °C (Table 3, entry 28) was used, or 52% of **1**, when the reaction was performed at room temperature (Table 3, entry 29). The latter experiment also required longer stirring time (2 days). Higher loads of the initiator, 50 and 100 mol% of LiBr (Table 3, entries 20 and 21) did not offer any improvement in the yield. Lithium iodide, unlike chloride and bromide, gave rather moderate yields (Table 3, entries 23 and 24) of **1**, in two different runs, 62% and 67%, respectively. Lower initiator loads, 5 mol%, (Table 3, entry 25), along with a decrease in the solvent volume to 5 mL (Table 3, entry 27) afforded **1** in much lower yields, 60% and 40%, respectively. This is consistent with the result obtained when the solvent volume was decreased to 5 mL (Table 3, entry 26) and **1** was produced in 56% yield. That suggests that under these conditions, significant cluster decomposition occurs. Surprisingly, the desired product can also be obtained in the absence of an initiator (Table 3, entry 30) but in rather moderate yield (40%). Diglyme was found to be inferior to tetrahydrofuran as the reaction solvent. The cluster **1** was afforded in only 50% yield (Table 3, entry 31) when the reaction was run at 100 °C but only the starting material was recovered when the temperature was lowered to 66 °C (Table 3, entry 32).

Our previous work involving dichlorocarbene generated from chloroform indicated that excess sodium hydride was required for the reaction to proceed. Our new method uses only 3.5 equivalents of NaH. The first equivalent is needed to convert the NMe_3_H^+^ salt of **12** to its Na^+^ salt and the second is needed to convert **12** to the dianion **11**. An excess of NaH, used as insurance against any adventitious moisture or carbon dioxide, is filtered off. In three parallel experiments, run in tetrahydrofuran, 1,2-dimethoxyethane and diglyme, excess sodium hydride (6 equivalents total) was not filtered off. All three experiments produced the product but they required three days to complete (Table 3, entries 33–35). This is consistent with recently published results [19] where excess sodium hydride is used and is not filtered off and the experiments are reported to be complete in nearly 70 h. A larger scale experiment, using 5 g of [NHMe_3_][B_11_H_14_], gave **1** in 77% yield (Table 3, entry 37), proving that the reaction is scalable.

It appears that contrary to our initial impression, the presence of the lithium cation is not necessary for the reaction to proceed readily. Other halide anion sources were examined as initiators for the generation of difluorocarbene from TMS-CF_3_. Alkali metal halides and alkaline earth metal halides (Table 4, entries 1–5 and 7–10) all afforded **1** in good and very good yields. Interestingly, the use of zinc chloride only led to cluster decomposition and no desired product (Table 4, entry 6). The desired **1** was obtained even when less usual halides were employed as the initiators. Palladium (II) chloride, gadolinium (III) fluoride and indium (III) chloride were effective in producing **1** (Table 4, entries 11–13). However, the experiments required two days to run to completion and the yields were not improved.

Other sources of dihalocarbenes, analogous to TMS-CF_3_, were also examined. When TMS-CCl_3_ and TMS-CBr_3_ were employed using the standard procedure (Table 5, entries 1, 2 and 5), no product was produced, suggesting that an additional equivalent of a base, expected from the stoichiometry, was necessary. When additional insoluble base, NaH, was present in the solution (Table 5, entries 4 and 7), the product **1** was produced in poor or trace quantities, respectively. However, when EtONa, a THF-soluble additional base, was present, **1** was formed but along with significant amounts of the [2-EtO-*closo*-1-CB_11_H_11_]^−^ byproduct (Table 5, entry 3), already familiar from earlier work in which CCl_2_ was generated from chloroform and base in the presence of EtONa [17]. TMS-CBr_3_ appeared to be less efficient than TMS-CCl_3_ in producing **1** even in the presence of EtONa and led to a mixture of products after prolonged heating (Table 5, entry 6). In contrast, known difluorocarbene sources, TMS-CF_2_Br and TMS-CF_2_Cl, did afford the desired **1** even in the absence of additional base but not in very good yields and the experiments required two days to complete (Table 5, entries 8 and 9).

### 2.2. Mechanism

The mechanism of dichlorocarbene insertion into [*nido*-B_11_H_14_]^−^ was investigated computationally before [17,22] and our results are similar. The results for difluorocarbene and dibromocarbene are new. 

**Figure 1:**
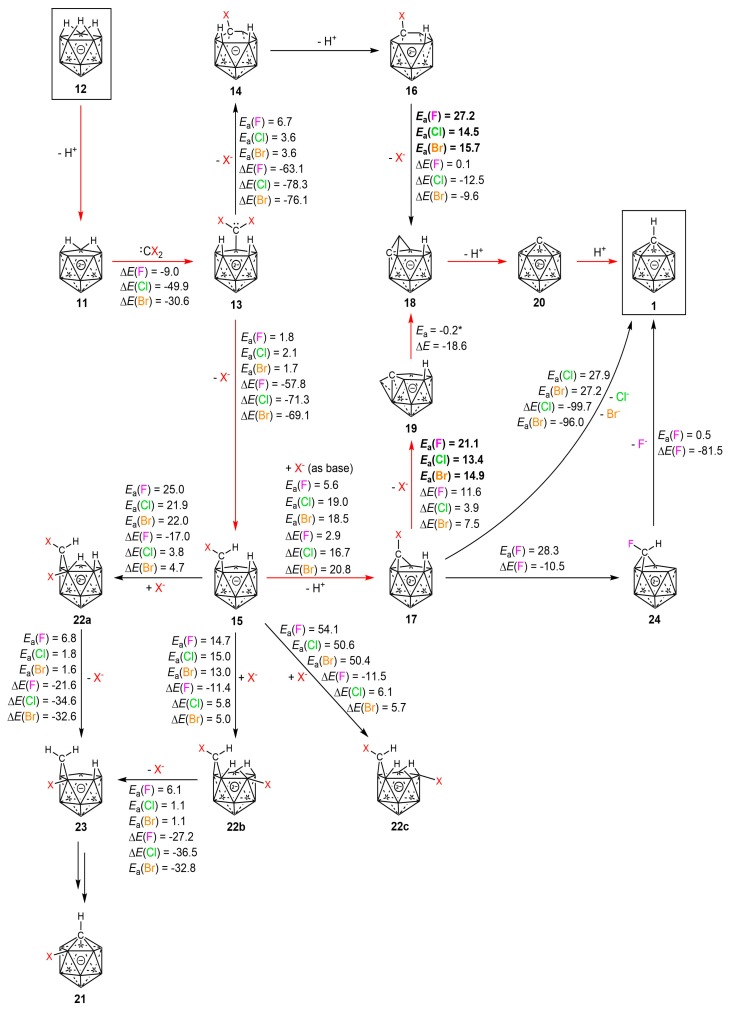
Calculated reaction energies and barriers for conversion of **12** to **1**. Bold: highest barrier in a branch. Red arrows: minimum energy path. Bottom left: pathways for halogenation in position 2. The -H^+^ sign: deprotonation by an added base (e.g. NaH or NaOEt). Asterisk at *E*_a_ (coversion of **19** to **18**): without zero-point vibrational energy, 0.01 kcal/mol.

In Figure 1, we present an updated and expanded calculation of possible pathways for the insertion of difluorocarbene, dichlorocarbene and dibromocarbene. After proton abstraction from **12**, the resulting **11** is attacked by the dihalocarbene, resulting in a barrierless formation of **13**. After the dianion **13** loses a halide, a branching of the reaction path occurs, leading to **14** (upper pathway, E_a_(F) = 6.7; E_a_(Cl) = 3.6; E_a_(Br) = 3.6 kcal/mol) and to **15** (lower pathway, E_a_(F) = 1.8; E_a_(Cl) = 2.1; E_a_(Cl) = 1.7 kcal/mol). Compound **14** can undergo deprotonation to form the dianion **16**, which could lose the second halide in a process that involves a high barrier in case of F (27.2 kcal/mol) and a lower one for Cl and Br (14.5 and 15.7 kcal/mol, respectively) to form **18**.

In the preferred lower pathway, compound **15** can undergo halogenation (eventually producing the side product **21**) or deprotonation (resulting in **17** and subsequently leading to **1**). The attack of the halide nucleophile at a boron vertex that is a next nearest neighbor to carbon is the most probable, and results in **22b**. The barriers are similar for both F and Cl (∼15 kcal/mol) and slightly lower for Br (13 kcal/mol).

Formation of **17** by deprotonation of **15** is interesting—in the anhydrous medium, fluoride (F^−^) can act as a base, easily (5.6 kcal/mol barrier) abstracting a hydrogen from **15**. The deprotonation by chloride and bromide has a much higher barrier (19.0 and 18.5 kcal/mol, respectively) and a significantly less favorable equilibrium (16.7 and 20.8 kcal/mol for Cl^−^ and Br^−^, respectively, vs. 2.9 for F^−^), implying that the reaction of **15** and Cl^−^ or Br^−^ is more likely to result in the formation of the halogenated side-product **22b**. Since chloride and bromide do not deprotonate **17**, the presence of an additional equivalent of strong base is required (NaH or preferably the soluble EtONa). 

In **17**, the transfer of the final bridged hydrogen to the carbon and the dissociation of the halide could result in the immediate formation of **1** but this reaction has relatively high barriers (28 kcal/mol in both cases). Instead, the halide can leave, which results in the carbon displacing a BH group from the upper pentagon to the side of the cage and forming **19.** This reaction has the highest barrier on the minimum energy path that leads to **1** (21.1 kcal/mol for F, 13.4 for Cl and 14.9 for Br). Compound **19** virtually immediately rearranges to **18**, which transforms to **20** by deprotonation (which closes the cage), followed by protonation resulting in the final product **1**.

## 3. Discussion

Two related mechanistic aspects of this primarily synthetic study appear noteworthy to us. First, not counting the base needed to convert the initial NMe_3_H^+^ salt of **12** into its sodium salt, only one equivalent of base, used to convert **12** to **11**, is needed for the reaction with CF_2_. Yet, at first sight stoichiometry suggests that two will be necessary to neutralize the HF formed: B_11_H_14_^−^ + CF_2_ = CB_11_H_12_^−^ + 2HF. Removing possibly present excess NaH from **11** by filtration does not hinder the reaction with difluorocarbene in the least and it appears that a large excess of NaH actually hinders it. Reluctantly, we conclude that one equivalent of HF is eliminated spontaneously and absorbed in the glassware. Otherwise stated, in the anhydrous environment the fluoride anion is acting as a base. In contrast, the less basic chloride and bromide anions do not and work with CCl_2_ and CBr_2_ requires additional base.

The second noteworthy observation is that against our original expectations based on the known affinity of fluorine for boron, difluorocarbene is greatly superior to other halocarbenes. We were initially reluctant to even try it for fear that one or both fluorine atoms will end up on boron vertices. In actual fact, this is not observed at all and CF_2_ is vastly superior to both CCl_2_ and CBr_2_. We attribute the difference to the ability of the fluoride anion to act as a strong base. This view is supported by the density functional theory (DFT) results for the conversion of **15** to **17** (Figure 1), which document the role that the much higher strength of the HF bond compared to the HCl and HBr bonds plays in permitting the removal of a proton from the cluster anion. When the reaction is performed with CCl_2_ or CBr_2_ instead of CF_2_, **15** is much more easily diverted toward other products, especially those with a nucleophile in position 2. This type of byproduct has not been observed at all when CF_2_ is used.

## 4. Materials and Methods 

### 4.1. General Information

Tetrahydrofuran, 1,2-dimethoxyethane and diglyme were dried and distilled from sodium/benzophenone under Ar. Reagents were used as supplied without purification unless stated otherwise. All operations were performed under argon using Schlenk techniques unless stated otherwise. Sodium hydride (60% oil dispersion) was washed with hexane three times under Ar before use. (Trifluoromethyl)trimethylsilane was purchased from Oakwood Chemical (Estill, SC, USA). (Difluoromethyl)trimethylsilane [23], (bromodifluoromethyl)trimethylsilane [24], (chlorodifluoromethyl)trimethylsilane [25], (tribromomethyl)trimethylsilane [26] and (trichloromethyl)trimethylsilane [27] were prepared according to reported procedures. ^1^H, ^11^B and ^19^F NMR spectra were recorded on Bruker Avance-III 300 MHz instrument in CDCl_3_ or CDC_3_N. Chemical shifts are reported in ppm.

### 4.2. Calculations

All density functional theory (DFT) calculations were done using the B3LYP functional, which has previously been used for the description of carborane reactions [22,28]. Long-range interactions were corrected using Grimme’s dispersion scheme with Becke-Johnson damping (D3BJ) [29]. Solvent effects were modelled using Truhlar’s SMD implicit solvent model [30]. All optimizations were followed by harmonic frequency calculations and all reported numbers include zero-point vibrational energy. Gaussian09B01 [31] was used for all calculations.

### 4.3. Procedures

**Figure 2:**
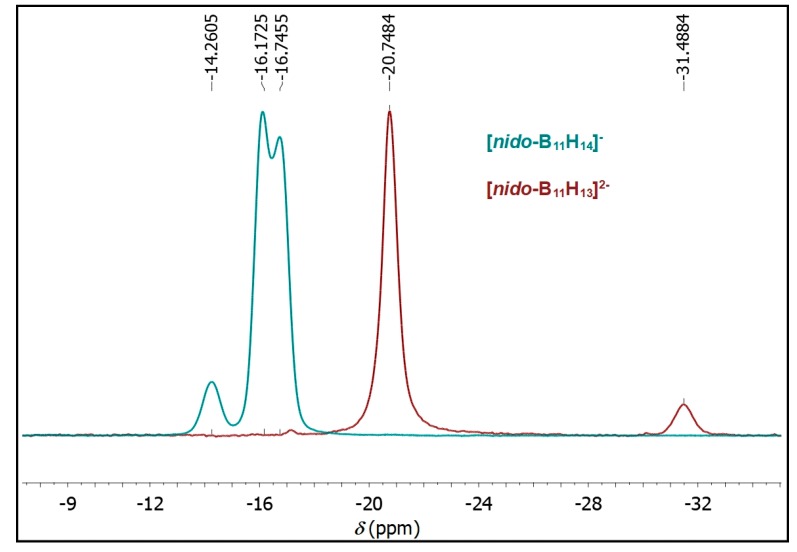
^11^B nuclear magnetic resonance (NMR) spectrum of [*nido*-B_11_H_14_]^−^ and [*nido*-B_11_H_13_]^2−^ in CD_3_CN.

*General procedure for preparation of [NEt_4_][CB_11_H_12_]* (**1**). A solution of [NHMe_3_][B_11_H_14_] [18] (250 mg, 1.29 mmol) in freshly-distilled THF (5 mL) was added dropwise to a suspension of NaH (109 mg, 4.53 mmol) in THF (5 mL) cooled to −40 °C under Ar. The reaction mixture was stirred at −40 °C for 15 min, allowed to warm to room temperature and stirred for 30 min. Gas evolution was observed during warming up. The formation of the dianion, [2Na][B_11_H_13_], was confirmed by ^11^B-NMR (Figure 2): ^11^B{^1^H} NMR (96 MHz, CD_3_CN) *d* −20.8 (10B), −31.5 (1B). The solution was then filtered through a cotton plug under Ar into an oven-dried flask charged with carefully dried LiCl (8.23 mg, 0.194 mmol, 15 mol%). The reaction mixture was heated to reflux and (trifluoromethyl)trimethylsilane (460 mg, 3.24 mmol, 0.48 mL) was added dropwise to the reaction flask. The solution turned yellow and then orange. The reaction mixture was refluxed for 16 h, cooled to room temperature and quenched with water (2 mL). The solvents were evaporated under reduced pressure, water (25 mL) was added to the residue and the solution was acidified with 10% aqueous HCl. The aqueous layer was extracted with Et_2_O (3 × 30 mL). The combined organic layers were transferred to a flask, water (30 mL) was added and the organic solvent was removed under reduced pressure. The resulting aqueous solution was filtered through a cotton plug and treated with solid NEt_4_Br (408 mg, 1.94 mmol). The precipitate was filtered and dried to give 276 mg (78% yield) of [NEt_4_][CB_11_H_12_] as a white solid. Alternatively, solid NHMe_3_Cl (186 mg, 1.94 mmol) can be used to yield [NHMe_3_][CB_11_H_12_]. Traces of impurities can be removed by recrystallization from EtOH/H_2_O: ^11^B-NMR (96 MHz, CD_3_CN) *d* −6.9 (d, *J* = 140 Hz, 1B), −13.3 (d, *J* = 135 Hz, 5B), −16.2 (d, *J* = 152 Hz, 5B). 

*Preparation of [NHMe_3_][B_11_H_14_]* (**3**). The salt of the anion was prepared according to a modified literature protocol [18]. The experimental setup consisted of a 500-mL three-neck flask equipped with an addition funnel, stopper and bent distillation adapter connected to a dry ice condenser. The flask was charged with NaBH_4_ (15 g, 0.397 mol) under Ar and freshly distilled diglyme (120 mL) was added resulting in a cloudy solution. With constant stirring, the mixture was heated to 105 °C and BF_3_ ·Et_2_O (72.0 g, 0.51 mol, 62.7 mL) was added dropwise (20 mL/h). When the addition was complete, heating was continued for 1 hr and the reaction mixture was allowed to cool to room temperature. The resulting yellow suspension was filtered in air and the solid was washed twice with dry diglyme (2 × 30 mL). Combined diglyme solution was evaporated under reduced pressure and the resulting yellow oil was treated with a solution of NHMe_3_Cl (6.69 g, 0.07 mol) in H_2_O (100 mL). The aqueous solution was extracted with dichloromethane (3 × 50 mL), the combined organic layers were dried (MgSO_4_) and evaporated to dryness. The resulting tacky solid was washed with Et_2_O (2 × 50 mL) and then suspended in H_2_O (100 mL). The suspension was stirred for 15 min, filtered and the solid was washed with H_2_O (50 mL) followed by Et_2_O (50 mL) and then dried in air to give 5.86 g of [NHMe_3_][B_11_H_14_] as a white solid (65% yield): ^1^H-NMR (300 MHz, CD_3_CN) *d* br m 0.4–2.5 (11H), 2.82 (s, 9H); ^11^B{^1^H} NMR (96 MHz, CD_3_CN) *d* −14.2 (1B), −16.1 (5B), −16.7 (5B).

## 5. Summary

We have developed a simple and efficient method for the preparation of the [*closo*-1-CB_11_H_12_]^−^ cluster anion from cheap and commonly available reagents, NaBH_4_, BF_3_ · Et_2_O, TMS-CF_3_ and NaH in only two steps in a 45–60% overall yield. Unlike other published protocols, our method requires only a simple experimental setup and commonly available glassware and it does not require toxic and expensive decaborane, excess reagents or glove-box and pressure vessels. Manual operations are simple and can be easily performed in a hood and the process is easily scalable. Moreover, in contrast to other methods, the product is afforded in just 16 hours. We have performed DFT calculations to develop a mechanistic proposal for the carbene insertion process and to account for the remarkable differences between various carbenes.

## Figures and Tables

**Table 1:** Carbene insertion into **11** ^a^.

Entry	Carbene Source	Solvent	Volume [mL]	Temp [°C]	Yield of 1 [%]
1	CHCl_2_P(O)(OEt)_2_	THF	20	rt	65
2	CHCl[P(O)(OEt)_2_]_2_	THF	20	rt	50 ^b^
3	CN_2_[P(O)(OEt)_2_]_2_	THF	20	rt	0 ^c^
4	CCl_3_COONa	Diglyme	10	150	0 ^c^
5	ClF_2_COONa	Diglyme	10	165	0 ^c^
6	CF_3_COOLi	Diglyme	10	165	0 ^c^

^a^ Experiments run on 250 mg of [NHMe_3_][B_11_H_14_]; with 2.5 equiv of carbene source. ^b^ Combined yield of **1** and [*nido*-7-OH-B_11_H_13_]^−^. ^c^ Boron cluster decomposition.

**Table 2:** Optimization of the reaction conditions using TMS-CF_3_ as the source of carbene ^a^.

No.	TMS-CF_3_ (equiv)	Solvent	Volume [mL]	Initiator [mol%]	Temp [°C]	Yield of 1 [%]
1	2.5	DME	20	TBAB [5]	85	67
2	2.5	DME	10	TBAB [5]	85	0 ^b^
3	2.5	THF	20	TBAB [5]	66	78
4	2.5	THF	10	TBAB [5]	66	75
5	4.0	DME	20	TBAB [5]	85	85
6	2.5	Diglyme	10	TBAB [5]	120	0 ^b^
7	4.0	DME	10	TBAF [5]	85	0 ^b^
8	2.5	DME	10	TBAB [5]	70	0 ^c^

^a^ Experiments run on 250 mg of [NHMe_3_][B_11_H_14_]. ^b^ Starting material was recovered. ^c^ [*nido*-7-OH-B_11_H_13_]^−^ was the major product; TBAB = tetrabutylammonium bromide; TBAF = tetrabutylammonium fluoride, 1.0 M in THF.

**Table 3:** Carbene insertion into **11** using TMS-CF_3_ as the carbene source ^a^.

No.	TMS-CF3 (equiv)	Solvent	Volume [mL]	Initiator [mol%]	Temp [°C]	Yield of 1 [%]
1	2.5	THF	10	LiCl [10]/TBAB [5]	66	94 ^b^
2	2.5	DME	10	LiCl [10]/TBAB [5]	85	0 ^c^
3	2.5	DME	10	LiCl [10]/TBAB [10]	70	0 ^c^
4	2.5	THF	20	LiCl [10]/TBAB [5]	66	76
5	2.5	DME	20	LiCl [10]/TBAB [5]	85	0 ^c^
6	2.5	THF	10	LiCl [10]/TBAB [10]	66	67 ^d^
7	3.5	THF	10	LiCl [10]/TBAB [5]	66	68
8	2.5	THF	10	LiCl [10]/TBAB [5]	66	69 ^e^
9	2.5	THF	10	LiCl [10]/TBAB [5]	60	61
10	2.5	THF	10	LiBr [10]/TBAB [5]	66	62
11	2.5	THF	10	LiI [10]/TBAB [5]	66	50
12	3.5	THF	10	LiBr [10]/TBAB [5]	66	65
13	5.0	THF	10	LiCl [10]/LiBr [10]	66	76
14	2.5	THF	10	LiCl [15]	66	78 ^f^
15	5.0	THF	10	LiCl [15]	66	78
16	5.0	THF	10	LiCl [25]	66	68
17	5.0	THF	10	LiCl [50]	66	73
18	2.5	THF	10	LiBr [15]	66	72
19	3.5	THF	10	LiBr [15]	60	45
20	3.5	THF	10	LiBr [50]	66	74
21	3.5	THF	10	LiBr [100]	66	77
22	3.5	THF	20	LiBr [15]	66	61
23	2.5	THF	10	LiI [15]	66	62
24	5.0	THF	10	LiI [15]	66	67
25	2.5	THF	10	LiCl [5]	66	60
26	2.5	THF	5	LiCl [15]	66	56
27	2.5	THF	5	LiCl [5]	66	40
28	2.5	THF	10	LiCl [15]	50	54
29	2.5	THF	10	LiCl [15]	rt	52 ^g^
30	2.5	THF	10	-	66	40
31	2.5	Diglyme	10	LiCl [15]	100	50
32	2.5	Diglyme	10	LiCl [15]	66	0 ^h^
33	2.5	THF	10	LiCl [15]	66	77 ^i^
34	2.5	DME	10	LiCl [15]	85	52 ^i^
35	2.5	Diglyme	10	LiCl [15]	100	25 ^i^
36	2.5	THF	10	LiCl [15]	66	56 ^j^
37	2.5	THF	200	LiCl [15]	66	77 ^k^

^a^ Experiments run on 250 mg of [NHMe_3_][B_11_H_14_]. ^b^ Two more runs gave 78% yield. ^c^ Decomposition of starting material. ^d^ Based on three runs. ^e^ 500 mg of [NHMe_3_][B_11_H_14_] was used. ^f^ Based on three runs. ^g^ Reaction mixture stirred for 2 days. ^h^ Starting material recovered. ^i^ Six equiv. of NaH, no filtration, stirred for three days. ^j^ Solvent evaporated after deprotonation, residue redissolved in THF. ^k^ 5.0 g of [NHMe_3_][B_11_H_14_] was used. TBAB = tetrabutylammonium bromide.

**Table 4:** Optimization of the reaction conditions using various metal halides as initiators and TMS-CF_3_ as the carbene source ^a^.

No.	TMS-CF_3_ (equiv)	Solvent	Volume [mL]	Initiator [mol%]	Temp [°C]	Yield of 1 [%]
1	2.5	THF	10	KF [15]	66	53
2	2.5	THF	10	NaCl [15]	66	65
3	2.5	THF	10	KCl [15]	66	54
4	2.5	THF	10	CsCl [15]	66	71
5	2.5	THF	10	MgCl_2_ [15]	66	64
6	2.5	THF	10	ZnCl_2_[15]	66	0 ^b^
7	2.5	THF	10	NaI [15]	66	60
8	2.5	THF	10	KI [15]	66	70
9	2.5	THF	10	CsI [15]	66	57
10	2.5	THF	10	KBr [15]	66	54
11	2.5	THF	10	PdCl_2_ [15]	66	76 ^c^
12	2.5	THF	10	GdF_3_ [15]	66	59 ^c^
13	2.5	HF	10	InCl_3_ [15]	66	65 ^c^

^a^ Experiments run on 250 mg of [NHMe_3_][B_11_H_14_]. ^b^ Cluster decomposition. ^c^ Reaction mixture was stirred for two days.

**Table 5:** Other dihalocarbene sources ^a^.

No.	Reagent (equiv)	Solvent	Volume [mL]	Initiator	Temp [°C]	Yield of 1 [%]
1	TMS-CCl_3_ (2.5)	THF	10	LiCl	66	0 ^b^
2	TMS-CCl_3_ (2.5)	diglyme	10	LiCl	120	0 ^b^
3	TMS-CCl_3_ (2.5)	THF	10	LiCl	66	21 ^c,d,e^
4	TMS-CCl_3_ (3.5)	THF	10	LiCl	66	5 ^c,f,g^
5	TMS-CBr_3_ (2.5)	THF	10	LiCl	66	0 ^b^
6	TMS-CBr_3_ (2.5)	THF	10	LiCl	66	40 ^c,d,h^
7	TMS-CBr_3_ (3.5)	THF	10	LiCl	66	Traces ^c,f,i^
8	TMS-CF_2_Br (2.5)	THF	10	LiCl	66	71 ^c^
9	TMS-CF_2_Cl (2.5)	THF	10	LiCl	66	47 ^c^

^a^ Experiments run on 250 mg of [NHMe_3_][B_11_H_14_]; 15 mol% of LiCl. ^b^ Starting material was recovered. ^c^ Reaction mixture stirred for two days. ^d^ Two equiv. of EtONa added. ^e^ Also a 6% yield of [*closo*-2-EtO-1-CB_11_H_11_]^−^. ^f^ 4 eq. of NaH (no filtration). ^g^ 5% yield of **1** and 40% recovered **12**. ^h^ Approximately 40% **1**, 40% of [*closo*-2-EtO-1-CB_11_H_11_]^−^ and 20% recovered **12**. ^i^ Traces of **1** and 35% recovery of **12**.
